# Nonoperative Treatment of Distal Biceps Brachii Musculotendinous Partial Rupture: A Report of Two Cases

**DOI:** 10.1155/2013/970512

**Published:** 2013-07-17

**Authors:** I. López-Zabala, J. A. Fernández-Valencia

**Affiliations:** Department of Orthopaedic Surgery, Hospital Clinic, 08036 Barcelona, Spain

## Abstract

Musculotendinous ruptures of the distal biceps brachii are extremely rare injuries whose clinical presentation is similar to distal biceps avulsion. We describe two cases of patients who suffered a distal biceps brachii musculotendinous partial rupture. The first patient was playing soccer as goalkeeper and experienced sudden pain while throwing the ball overhead with his left arm. The second patient experienced sudden pain while weightlifting with his right arm. The mechanism of injury was the same in the two cases, as both involved glenohumeral elevation with elbow extension and forearm supination. Neither of these two patients underwent surgical repair or rehabilitation, and both had perfect scores of 100 on the Mayo Clinic Performance Index for the Elbow at one-year followup.

## 1. Introduction

Musculotendinous ruptures are common injuries among athletes [1–3]. However, distal biceps brachii musculotendinous ruptures in particular are very rare, and few references exist in the literature [4]. In contrast, distal biceps brachii avulsion from the radial tuberosity is a common injury [5–9] that can be mistaken for a musculotendinous rupture [4]. The aim of this paper is to present two cases of distal biceps brachii musculotendinous ruptures, the results obtained with nonsurgical treatment, and a review of the literature.

## 2. Case Reports

The first patient was a fifty-one-year-old male without significant associated comorbidities who consulted because of acute pain in his left arm in April of 2007. He described having experienced a sharp and sustained pain as he threw a ball overhead with his left arm while playing soccer as goalkeeper. Physical examination showed pain on mobilization of the elbow and tenderness in the distal anterior arm with no distal sensory disturbances. Distal radial and ulnar pulses were preserved. Study by ultrasound confirmed the partial rupture of the biceps brachii at its distal myotendinous junction with preservation of the tendon. The patient was prescribed oral analgesic therapy, his limb was immobilized with a sling, and daily assisted active mobilization was encouraged. The sling was removed after 3 weeks, allowing active mobilization Rehabilitation was not indicated and the patient resumed his previous work and sports activities. After one year, the patient could perform complete flexoextension and pronosupination; despite a subjective slight decrease of strength in comparison to his pre-injury status, he experienced no difficulty in performing daily life activities or engaging in sports and scored a perfect score of 100 on the Mayo Elbow Performance Score (MEPS).

**Figure 1:**
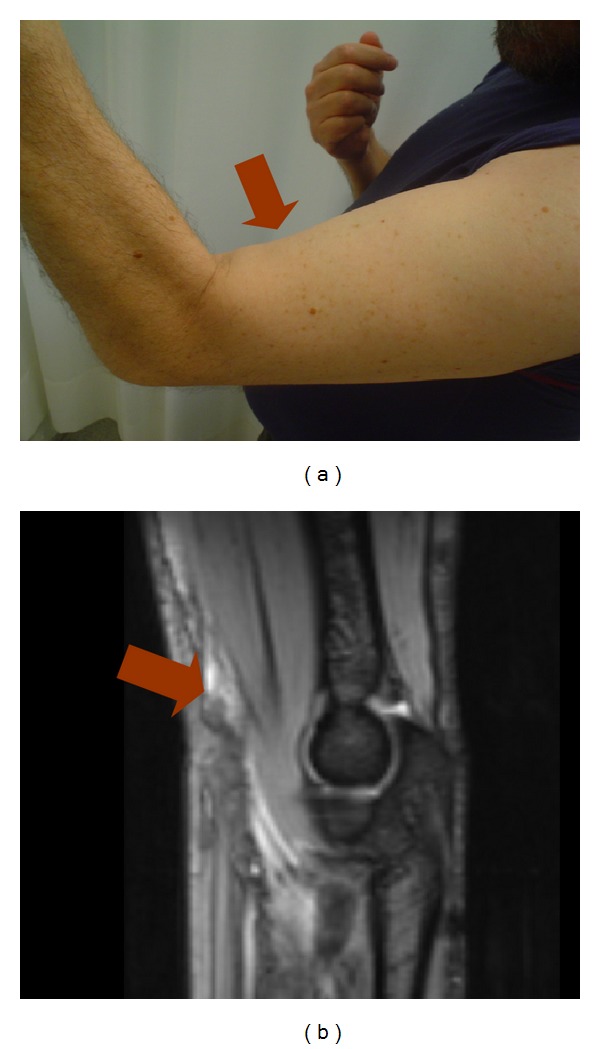
Clinical and MRI image of the myotendinous rupture of the biceps brachii muscle.

**Figure 2:**
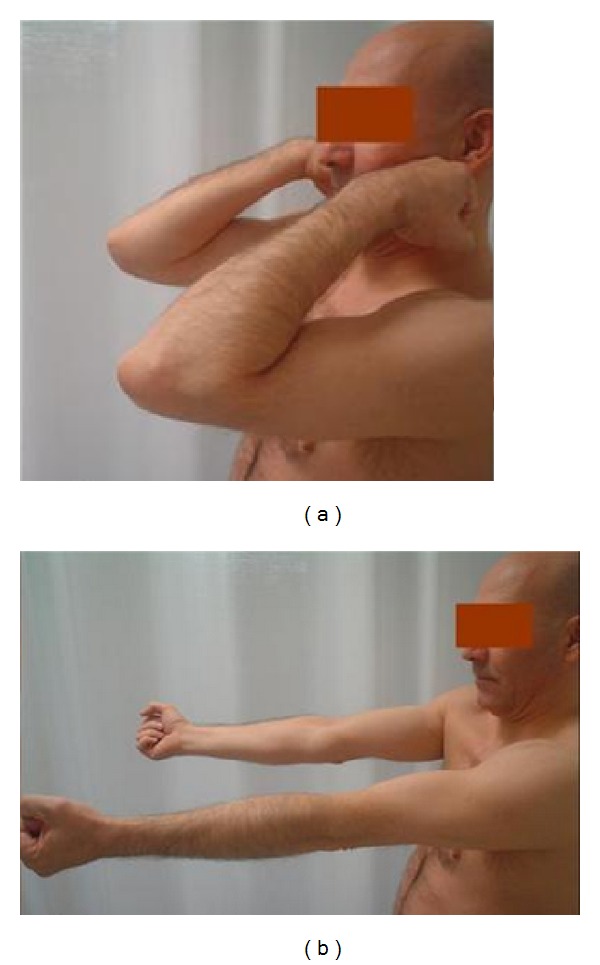
Appearance after one year with complete flexion and extension of the elbow.

The second patient was a forty-eight-year-old male, without significant associated comorbidities who experienced a sudden onset of severe pain in his right arm while weightlifting in November of 2007. Physical examination revealed an anterior depression of his arm, tenderness in the anterior and distal arm, and inability to perform supination. No associated neurological or vascular deficits were observed. A magnetic resonance imaging (MRI) was performed which confirmed the presence of a partial rupture of the biceps brachii muscle in the distal myotendinous junction with preservation of the tendon (Figure 1). The patient was prescribed oral analgesic therapy, his limb was immobilized with a sling, and daily assisted active mobilization was encouraged. The sling was removed after 3 weeks, allowing active mobilization. Rehabilitation was not indicated and the patient resumed his activities gradually. At one-year followup the patient was able to perform complete flexionextension and full supination (Figure 2). As with the first case, the patient reported a slight subjective decrease of strength in comparison to his pre-injury status but experienced no difficulties in performing daily life activities or playing golf again. His MEPS score was also 100.

## 3. Discussion

Injuries of the distal biceps brachii tendon are common and are most often avulsions of the insertion of the biceps brachii tendon. Although the diagnosis and treatment of this last entity have been widely studied, the diagnosis and treatment of musculotendinous junction ruptures have been scarcely reported in the literature [4].

The only series that we found belongs to Schamblin and Safran [4] with 6 cases. According to Morrey [5], the injury is more likely to occur among individuals who suffer encephalopathy. This condition was not observed in any of the cases reported by Schamblin and Safran [4] who did, however, note that three of the six cases had a history of recreational weightlifting. Likewise, neither of our two cases reported a history of encephalopathy. In both cases the mechanism of injury was glenohumeral elevation with elbow extension and forearm supination [5]. Schamblin and Safran [4] showed good and excellent results after three years of followup without surgical treatment (5 cases scored 100 and 1 scored 85 on the MEPS). With regard to treatment, the authors indicated that a nonsurgical approach does not imply “doing nothing,” but rather that in order to achieve satisfactory functional recovery the patient should perform directed physical therapy. In the two cases presented here, both patients were in their fifties and engaged in sports recreationally, neither engaged in physical therapy, and yet both were satisfied with the outcome. 

In conclusion, further studies are needed to better understand the epidemiology of this entity, its pathogenesis, and possible need for surgery. Based on the results obtained in our two cases, we suggest that people who engage in recreational sports and suffer a rupture of the distal biceps brachii musculotendinous junction can achieve satisfactory functional results without either surgical treatment or directed physical therapy.
